# Protective effect of brain microvascular endothelial cell-derived exosomes on blood-brain barrier induced by ischemia-reperfusion injury *in vivo* and *in vitro*

**DOI:** 10.3389/fphar.2025.1548122

**Published:** 2025-04-29

**Authors:** Jin Sun, Meng Wang, Lichen Guo, Yushuang Cao, Linlin Su, Shaoxia Wang, Lijuan Chai, Qing Yuan, Limin Hu

**Affiliations:** ^1^ State Key Laboratory of Chinese Medicine Modernization, Tianjin University of Traditional Chinese Medicine, State Key Laboratory of Component-Based Chinese Medicine, Ministry of Education Key Laboratory of Pharmacology of Traditional Chinese Medicine Formulae, Tianjin Key Laboratory of Traditional Chinese Medicine Pharmacology, Tianjin University of Traditional Chinese Medicine, Tianjin, China; ^2^ School of Medical Technology, Tianjin University of Traditional Chinese Medicine, Tianjin, China

**Keywords:** ischemic stroke, exosomes, cerebral microvascular endothelial cells, pericytes, blood-brain barrier, middle cerebral artery occlusion/reperfusion

## Abstract

**Background:**

Enhanced blood-brain barrier (BBB) permeability exacerbates clinical symptoms and long-term disability after ischemic stroke. Exosomes derived from cerebral microvascular endothelial cells (EC-Exo) can enhance neural function recovery in MCAO/R mice. However, it remains unclear whether the brain protective effects of EC-Exo are associated with improved BBB structure and functionality.

**Methods:**

This study developed an *in vitro* BBB model by co-culturing endothelial cells (bEnd.3) with pericytes (MBVP) to examine the effects of EC-Exo on BBB integrity. The neurobehavioral function of EC-Exo was evaluated *in vivo* using the rotarod test and gait assessment. The permeability of BBB was evaluated using the Evans blue penetration test and IgG leakage test. The integrity of the BBB structure was assessed using immunofluorescence and Western blot analysis. Mechanistic investigations aimed to elucidate the regulatory role of PDGF-PDGFRβ and Ang1/Ang2-Tie2 pathways in maintaining BBB integrity.

**Results:**

EC-Exo improves BBB integrity by increasing TEER values and decreasing Papp *in vitro*. Besides, EC-Exo not only reduces gait abnormalities in MCAO/R-injured mice, attenuates BBB permeability *in vivo*. EC-Exo enhances the expression of tight junction and basement membrane proteins. Mechanistic studies have demonstrated that EC-Exo can effectively activate the PDGF-PDGFRβ and Ang1/Ang2-Tie2 signaling pathways, thereby facilitating the maintenance of BBB integrity, and these effects were verified with PDGFRβ inhibitor and Tie2 inhibitor *in vitro*.

**Conclusion:**

In conclusion, EC-Exo enhances BBB integrity by activating PDGF-PDGFRβ and Ang1/Ang2-Tie2 signaling pathways, promoting communication between endothelial cells and pericytes. This introduces an innovative adjuvant therapy for treating ischemic stroke.

## 1 Introduction

Stroke is a severe neurological condition linked to significant mortality and disability (Mukherjee and Patil, 2011). Stroke is mainly divided into ischemic and hemorrhagic, of which ischemic accounted for about 71% of the total number of strokes (Roger et al., 2012). Blood-Brain Barrier (BBB) dysfunction is a clinical pathological characteristic of ischemic stroke (Candelario-Jalil et al., 2022). The BBB consists of tightly connected vascular endothelial cells, an intact basal membrane, pericytes, and a glial membrane encased by astrocyte foot plates (Yuan et al., 2021). Ischemic stroke leads to tight junction dysfunction and the activation of pericytes and astrocytes, causing irreversible capillary contraction and BBB damage (Shin et al., 2015). Promoting the structural and functional recovery of the BBB is an effective treatment for preventing and treating substantial disability in stroke patients.

The cerebral microvascular endothelium’s role in angiogenesis and maintaining barrier integrity is vital for prognosis after ischemia, making it a key therapeutic target post-stroke (Nitzsche et al., 2021). Pericytes are crucial for cerebrovascular development and are key to the formation and maintenance of the BBB. Pericytes play a vital role in maintaining BBB structural integrity during disease progression by secreting pro-angiogenic factors via coordinated endothelial pathways and signaling molecules (Hu et al., 2017; Cai et al., 2017). The interaction between these cell types is essential for maintaining BBB integrity after a stroke.

Exosomes (Exo), extracellular vesicles ranging in size from 30 to 200 nm, possess the capability to traverse the BBB (Pegtel and Gould, 2019). These exosomes contain proteins, nucleic acids, and lipids. They are crucial for intercellular communication under both normal and abnormal conditions by enabling cargo transport between donor and recipient cells (Van Niel et al., 2018). Research has demonstrated the beneficial role of extracellular vesicle-mediated cellular communication in the pathophysiological process of cerebral ischemia (Gao et al., 2020). Research has shown that exosomes from astrocytes enhance neural plasticity and aid functional recovery post-stroke in rats (Xin et al., 2017). Exosomes derived from cerebral microvascular endothelial cells (EC-Exo) protect SH-SY5Y nerve cells from ischemia/reperfusion injury (Xiao et al., 2017). EC-Exo are essential for restoring neuronal vascular units and preserving the brain after acute ischemic injury (Zhou et al., 2020). Previous studies have demonstrated that EC-Exo exhibits potential neuroprotective effects against stroke by facilitating synaptic remodeling and suppressing apoptosis (Sun et al., 2022). However, the precise biological role of EC-Exo in maintaining BBB integrity following stroke remains elusive.

This study assessed the effects of EC-Exo on barrier integrity using an *in vitro* co-culture model of endothelial cells and pericytes under Oxygen-glucose deprivation and reperfusion (OGD/R) conditions, and an *in vivo* mouse model of MCAO/R. These findings enhance the understanding of EC-Exo’s protective mechanism against ischemia-reperfusion injury.

## 2 Methods

### 2.1 Cell culture

We obtained mouse brain microvascular endothelial cells (bEnd.3, CRL-2299) from the American Type Culture Collection (ATCC, Manassas, VA, United States) and mouse brain perivascular cells (MBVP, 342,014) from the BeNa Culture Collection (BNCC, Beijing, China). A humidified environment with 5% CO_2_ was maintained at 37 °C for cell culture in DMEM (Gibco, CA, United States) with 10% fetal bovine serum (FBS) and 1% penicillin-streptomycin.

### 2.2 Exosome isolation and identification

EC-Exo was isolated and collected using the previously described ultracentrifugation method (Théry et al., 2006). The supernatant from bEnd.3 cell culture, after serum-free medium replacement, was collected and underwent sequential ultracentrifugation at 4 °C: 200 x g for 10 min, 2,000 x g for 20 min, 10,000 x g for 30 min, and 100,000 x g for 70 min to remove dead cells and debris. Maintaining stability and preventing degradation of exosomes was achieved at −80°C.

Analyze the particle size distribution of exosomes diluted in PBS by Malvern Instruments. Incubation at room temperature for 3 minutes was performed with exosomes resuspended in PBS placed on a copper grid with 2 nm pore sizes. Following this, the liquid was gently blotted dry using filter paper before negative staining with a 2.5% phosphotungstic acid solution for 10 min at room temperature. Exosome morphology and dimensions were analyzed using transmission electron microscopy (TEM) (Hitachi, Japan) and Dynamic light scattering (DLS) (England, Malvern Nano ZS).

### 2.3 Fluorescently labelled exosomes and cellular uptake

EC-Exo (5,000 μg/mL) were labeled with 1 μM CM-Dil dye (C7000, Thermo, United States) in PBS for 30 min at 37 °C, then centrifuged at 100,000 *g* for 2 h at 4°C to remove excess dye. Subsequently, the stained exosome precipitates were resuspended in 200 ul of PBS.

The labeled EC-Exo was incubated with MBVP cell culture medium at 37°C and 5% CO_2_ in darkness for 12 h refer to previous studies (Hu, et al., 2022). Cells were fixed with 4% PFA (#P0099, Beyotime, Shanghai, China) and stained with FITC Phalloidin (#40735ES75, Yeasen, Shanghai, China) for cytoskeleton visualization and DAPI for nuclear staining.

### 2.4 *In Vitro* OGD/R model

To simulate ischemia-like conditions *in vitro*, MBVP cells were subjected to OGD. The cells were rinsed thrice with PBS and subsequently cultured in DMEM basal medium devoid of serum and glucose (#11966–025, Gibco, NY, United States). Cells were incubated in a triple gas incubator (PHCbi, MCO-5M) with 5% CO_2_ and 95% N_2_ for 6 h to induce OGD. Subsequently, they were returned to standard conditions with regular medium for 18 more hours. Control cells were kept under standard conditions with regular medium for the same period.

### 2.5 Establishment of an *in vitro* BBB model

An *in vitro* BBB model was developed by co-culturing pericytes and endothelial cells, where MBVP cells were grown on inverted Transwell^®^ plates (PICM03050, Millipore, NY, United States) featuring a 0.4 µm pore polycarbonate membrane. We utilized a 1:1 ratio of bEnd.3 to MBVP as determined by prior optimization experiments (Yuan, et al., 2021). The cells were placed upside down in a CO_2_ incubator for 4 h to facilitate adherence to the membrane. Subsequently, bEnd.3 cells were grown on Transwell^®^ plate films by placing the Transwell chambers squarely in the corresponding culture plate wells. The cells were incubated for 24 h at 37°C with 5% CO_2_. The study comprised four experimental groups: the control group (bEnd.3/MBVP), the model group (bEnd.3/MBVP + OGD), the GW4869 group (bEnd.3 + GW4869/MBVP + OGD), and the EC-Exo group (EC-Exo + MBVP + OGD). Based on previous studies (Sun et al., 2022), the dosage of EC-Exo was 120 ug/mL.

### 2.6 *In vitro* assay for BBB permeability

BBB permeability post-OGD treatment was evaluated using transendothelial electrical resistance (TEER) and the sodium fluorescein (Na-Flu) permeability coefficient (Papp). Tight junction integrity was assessed through TEER measurements, utilizing an electronic circuit with EVOM2 and STX2 electrodes (World Precision Instruments, Sarasota, FL, United States) to measure cellular resistances. All TEER data were normalized against the cell-free insert value for each treatment group. In the Na-Flu permeability assay, 10 μg/mL Na-Flu (Sigma-Aldrich) was introduced into PBS, and the lower chamber was refreshed with new PBS following OGD exposure. Following a 2-h incubation period in darkness, the quantification of Na-Flu leakage was performed using a fluorescent multifunctional zymograph. The absorbance values were measured at 620 nm.

### 2.7 Crystal violet staining

After OGD treatment, the upper layer cells of the Transwell chamber were gently swabbed using a cotton swab. Subsequently, the chamber was washed twice with PBS to thoroughly cleanse both sides. The fixed cells were then treated with 4% PFA (#P0099, Beyotime, Shanghai, China). After fixation, cells were stained for 20 min at room temperature with 0.1% crystal violet solution (#C0121, Solarbio, Beijing, China), followed by PBS washing to remove excess dye. Five randomly selected fields of view were captured using an inverted phase contrast microscope to obtain images. Crystal violet was decolorized with 33% acetic acid (Sinopharm Chemical Reagent Co. Ltd., Beijing, China), and the eluent was transferred to a 96-well plate. Optical density (OD) was measured with an enzyme-labeled instrument at 570 nm.

### 2.8 MCAO/R model and exosome treatment

We obtained 48 male C57BL/6J mice from Beijing Viton Lever Laboratory Animal Technology Co. Ltd. in Beijing, China. The mice were maintained at 25°C with a 12-hour light-dark cycle and fed standard food and water. Animal experiments were done in accordance with the National Institute of Health’s Guide for the Care and Use of Laboratory Animals and approved by the Animal Care and Use Committee of Tianjin University of Traditional Chinese Medicine, Tianjin, China (permit No. TCMLAEC2021246, 14 November 2021).

Transient middle cerebral artery occlusion (tMCAO) was conducted using the intracanalicular vascular occlusion technique, as outlined in prior research 9 (Engel et al., 2011). Male C57BL/6J mice (21–25 g) were anesthetized using 3% isoflurane (#R510-22, Rivard, China) and maintained with 1.0% isoflurane in a mixture of 70% NO_2_ and 30% O_2_. Rectal measurements confirmed that body temperature was consistently maintained at 37.0 °C during the procedure and occlusion using a heating pad. The middle cerebral artery’s origin was occluded by inserting a nylon silicone-coated monofilament (0.20 ± 0.01 mm, A5-1,620, Sinon, China) with a rounded tip via the right external carotid artery into the internal carotid artery. The filament was extracted 90 min post-occlusion to initiate reperfusion. After 24 h of reperfusion, the neurobehavioral outcome of the mice was assessed using the Zea-Longa score. Neurological outcomes were evaluated utilizing a five-point scale (Belayev et al., 1996). Mice scoring 1–3 were deemed successful for MCAO/R, excluded from the study who scored 0 or 4. Mice with subarachnoid hemorrhage and those without observable neurological deficits following MCAO/R were excluded from further analysis. Based on prior research (Sun et al., 2022), mice received tail vein injections of either exosomes (60 μg of total protein in 100 μL PBS, n = 16) or PBS alone were administered (100 μL, n = 16) for 7 consecutive days.

### 2.9 Gait analysis

The DigiGait system, specifically designed for mice, was utilized to capture and analyze their gait patterns. Prior to the experiment, a three-day acclimation period was provided for the mice. Subsequently, adjustments were made to the viewing frame in order to ensure clear visibility of the mouse’s paw in the captured image. Gait measurements were recorded with the speed set to 15 cm/s. The video recording continued until the rat completed at least three uninterrupted runs, during which it maintained continuous movement without stopping, jumping, or placing its paw on the wall of the room. The acquired records were processed and analyzed using DigiGait imaging system software.

### 2.10 Rotarod test

The motor function of mice was assessed using a fatigue rotarod apparatus. The mice underwent 3 days of training prior to the surgery. The fatigue rotating rod test was conducted on mice at 3, 7 and 14 days post-operation. The mice were positioned on a stationary rod rotator, which was then accelerated to 40 revolutions per minute (r/min). The duration for which the mice could maintain their balance while walking on the rod within a span of 5 min was recorded.

### 2.11 Evans blue extraversion after MCAO and recanalization

The *in vivo* integrity of the BBB was assessed using Evans blue (EB) solution (Sigma, #E2129, United States). The mice were injected with 2% EB (4 mL/kg) through the tail vein before being euthanized. Following a 12-hour circulation period, 0.1 mol/L PBS was used for trans-cardiac perfusion, after which the mouse brain was subsequently sectioned into coronal slices with a thickness of 1 mm at a rapid pace. The ischemic brain tissue was then homogenized by introducing 200 μL of a 50% trichloroacetic acid solution to the aforementioned tissue. The mixture was centrifuged at 5,000 rpm for 10 min at 4 °C to obtain the supernatant. The spectrophotometer measured the extravasated EB in the sample at 632 nm.

### 2.12 Microvessel pericyte coverage index

The Microvessel Pericyte Coverage Index (MPI) (Deng et al., 2019) measures the extent of pericyte coverage on microblood vessels. Immunofluorescence double staining using CD31 and PDGFRβ was employed to label microvessels and pericytes, respectively. Five random visual fields within the ischemic penumbra area were selected under a high-power microscope. PDGFRβ-positive cells refer to pericytes connected to vessels marked with CD31, indicating the number of microvessels covered by pericytes as the Microvessel Pericyte Density (MPD) (Yamamoto, et al., 2014). By counting MPD and the total number of microvessels, MPI can be calculated using the formula: MPI = MPD/total number of microvessels × 100%.

### 2.13 Immunofluorescence (IF) and IgG staining

Frozen brain tissue sections were immune-stained after fixation in 4% paraformaldehyde at room temperature and rinsed three times with PBS. IF staining was conducted on 10 μm thick cryosections of mouse brain tissue. Brain sections were washed three times with PBS (5 min each) and then rinsed in 0.5% Triton X-100 (ST797, Beyotime, Shanghai, China) for 30 min at room temperature. After three 5-min PBS washes, brain sections were blocked with 5% goat serum (S9070, Solarbio, Beijing, China) for 2 h. The sections were then incubated overnight at 4°C in a moist chamber with primary antibodies: Anti-ZO1, Anti-Claudin5, Anti-Collagen IV, Anti-Laminin, Anti-Occludin and Anti-PDGFR-β. The following day, sections were incubated with corresponding secondary antibodies for 2 h. For IgG staining, a TRITC-labeled goat anti-mouse IgG antibody (1:300 dilution, #6921–100, BioVision, MA, United States) was applied and incubated at 37°C for 2 h in the dark, with 1×PBS solution serving as a negative control. The slices were treated sequentially with PBS washing, DAPI staining for nucleation, then it was examined by an inverted fluorescence microscopy.

### 2.14 Western blot analysis

Proteins from exosomes and animals were separated using SDS-PAGE. Electrophoretic transfer to polyvinylidene difluoride (PVDF) membranes (Millipore, MA, United States) was conducted overnight at 4°C using anti-Claudin5, anti-Occludin, anti-ZO-1, anti-Collagen IV, anti-PDGFBB, anti-PDGFR-β, anti-Ang1, anti-Ang2 and anti-Tie2. Samples were incubated with horseradish peroxidase-conjugated antibodies. The Western blot bands were conducted utilizing ECL solution (Millipore, #WBKlS0100, MA, United States). Image-Pro Plus 6.0 was utilized for the quantitative analysis of Western blot data. The detailed information regarding antibodies is presented in Table 1.

**TABLE 1:** Primary antibodies used for western blot and immunostaining.

Antibody	Company	Catalog number	Dilution
TSG101	Abcam (MA, United States)	ab125011	1:1,000 (WB)
CD9	Abcam (MA, United States)	ab92726	1:1,000 (WB)
CD63	Abcam (MA, United States)	Ab68418	1:1,000 (WB)
Calnexin	ABclonal (Wuhan, China)	A24433	1:1,000 (WB)
Claudin5	Bioworld (MA, United States)	BS1069	1:1,000 (WB) 1:500 (IF)
Occludin	Bioworld (MA, United States)	BS72035	1:1,000 (WB) 1:500 (IF)
ZO-1	Abcam (MA, United States)	ab221547	1:1,000 (WB) 1:500 (IF)
Collagen IV	Abcam (MA, United States)	ab19808	1:1,000 (WB) 1:500 (IF)
Laminin	Abcam (MA, United States)	ab11575	1:1,000 (WB) 1:500 (IF)
PDGFR-β	Abcam (MA, United States)	ab60506	1:1,000 (WB) 1:200 (IF)
PDGFBB	Abcam (MA, United States)	ab178409	1:1,000 (WB)
CD31	Abcam (MA, United States)	Ab9498	1:1,000 (IF)
Ang1	Proteintech (United States)	23302-1-AP	1:1,000 (WB)
Ang2	Proteintech (United States)	24613-1-AP	1:1,000 (WB)
Tie2	Proteintech (United States)	19157-1-AP	1:1,000 (WB)
β-actin	Cell Signaling (CA,United States)	4970S	1:1,000 (WB)

### 2.15 Statistical analysis

The sample data from all groups were analyzed using SPSS 23.0 and GraphPad Prism 8.0 software, which met the criteria for equal variance according to Levene’s test and normal distribution as per the Shapiro–Wilk test, with both showing significance at *P* < 0.05. Data analysis was conducted using one-way ANOVA with Tukey’s *post hoc* test for multiple comparisons. Nonparametric data were analyzed using Kruskal–Wallis ANOVA, followed by Dunn’s test for post-hoc comparisons when *P* < 0.05 indicated statistical significance.

## 3 Results

### 3.1 Isolation and identification of exosomes

**FIGURE 1:**
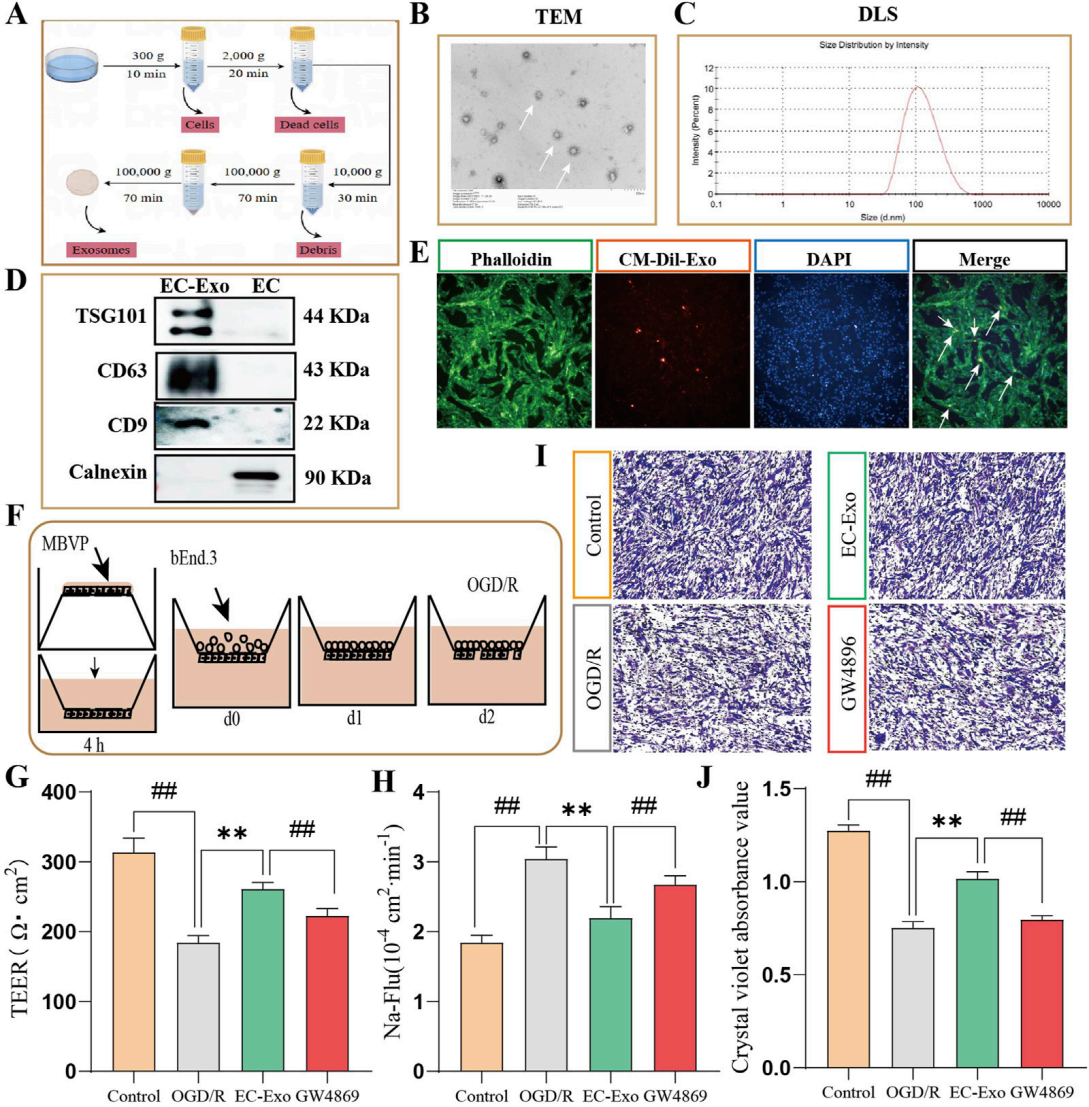
Extraction of exosomes and the effects on barrier integrity *in vitro* co-culture systems. **(A)** Extraction and purification process for exosomes from brain microvascular endothelial cells. **(B, C)** TEM was used to observe the microscopic morphology of EC-Exo. The nano-diameter range and average size of exosomes are determined using DLS. **(D)** Identifying the Exo marker proteins TSG101, CD63 and CD9 by Western blot. **(E)** Fluorescence microscopy confirmed the internalization of CM-Dil labeled exosomes by MBVP cells. **(F)** Diagram of co-culture of pericytes and endothelial cells. **(G)** The TEER values of EC-Exo on the barrier integrity of co-culture system (n = 9). **(H)** The permeability of Na-Flu (n = 9). **(I)** The dissociation of MBVP was evaluated by crystal violet staining (n = 4). **(J)** The quantitative analysis of dissociation coefficient (n = 4). The data were analyzed by one-way ANOVA followed by multiple comparisons using Tukey’s *post hoc* test. ^#^ *P* < 0.05, ^##^ *P* < 0.01 vs. control; ^*^ *P* < 0.05, ^**^ *P* < 0.01 vs. OGD/R.

EC-Exo were isolated from endothelial cell culture medium through differential ultrafast centrifugation and characterized using TEM, DLS and Western blot analysis (Figure 1A). The TEM analysis showed that EC-Exo had a spherical shape with diameters between 30 and 150 nm (Figure 1B). The DLS analysis demonstrated an average particle size of 89 nm for the exosomes (Figure 1C). Western blot analysis confirmed the presence of exosomal marker TSG101, CD63, and CD9 in EC-Exo, and the absence of non-exosomal marker Calnexin (Figure 1D).

### 3.2 The uptake of EC-Exo by MBVP cells

We examined MBVP cell uptake of EC-Exo by incubating them with CM-Dil labeled exosomes for 12 h. Fluorescence microscopy revealed cytoplasmic localization of CM-Dil-labeled exosomes (orange) within MBVP cells (green), indicating successful internalization of the exosomes by MBVP (Figure 1E).

### 3.3 EC-Exo maintained barrier integrity *in vitro*

The *in vitro* co-culture model of the BBB consisting of pericytes and endothelial cells illustrated in Figure F. The BBB’s integrity was evaluated by measuring the TEER and Papp for Na-Flu molecules *in vitro*. Results showed that OGD/R treatment notably decreased TEER values and elevated Na-Flu transmission in the co-culture BBB model compared to the control group. Conversely, treatment with EC-Exo significantly elevated TEER values (*P* < 0.05) and attenuated Na-Flu transmission (*P* < 0.05). However, the effects were reversed upon addition of EC-Exo inhibitors GW4869 (Figures 1G, H). The effect of EC-Exo on pericyte dissociation was evaluated using crystal violet staining. The findings indicated a notable rise in exfoliated MBVP cells after OGD/R. However, upon addition of EC-Exo, there was a reduction in shed cells and decreased pericyte dissociation. Notably, this effect was also inhibited by the introduction of EC-Exo inhibitor GW4869 (Figures 1I, J).

### 3.4 EC-Exo alleviated motor deficits in MCAO/R mice

**FIGURE 2:**
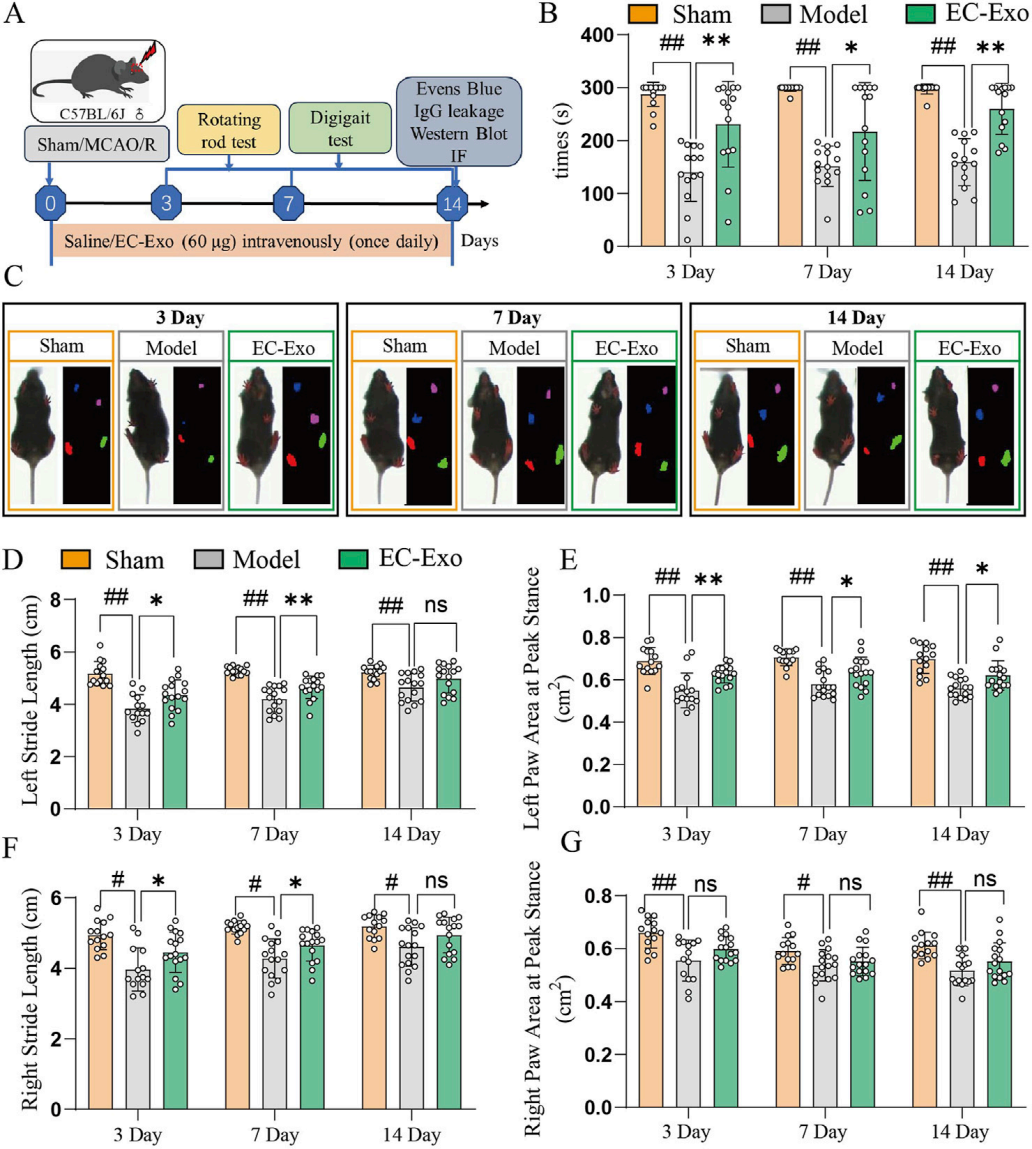
Assessment of post-stroke motor coordination and the integration of motor movement. **(A)** Design of animal experiments. **(B)** Times of in the rotarod in mice after MCAO/R (n = 14). **(C)** Gait analysis was performed on mice at 3, 7, and 14 days after MCAO/R. **(D)** Analysis of left stride length in mice at 3, 7, and 14 days post-MCAO/R (n = 14). **(E)** Analysis of the left paw area at peak stance in mice at 3, 7, and 14 days post-MCAO/R (n = 14). **(F)** Analysis of right stride length in mice at 3, 7, and 14 days post-MCAO/R (n = 14). **(G)** Analysis of the right paw area at peak stance in mice at 3, 7, and 14 days post-MCAO/R (n = 14). The data were analyzed by one-way ANOVA followed by multiple comparisons using Tukey’s *post hoc* test. ^#^ *P* < 0.05, ^##^ *P* < 0.01 vs. control; ^*^ *P* < 0.05, ^**^ *P* < 0.01 vs. MCAO/R.

The experimental design for the animal study is illustrated in Figure 2A. Due to brain damage, the mice showed more abnormal limb movement on the opposite side of the modeling side. In the rotarod test, mice treated with EC-Exo demonstrated a significant increase in latency to fall, indicating enhanced motor coordination and balance (Figure 2B). Gait analysis was conducted on mice after surgery to assess functional deficits resulting from MCAO/R at 3, 7, and 14 days. Gait stride length and Paw area at Peak Stance were selected to describe the gait (Figure 2C). After 14 days of MCAO/R, mice in the EC-Exo group demonstrated significant improvement in spatial parameters, unlike the model group, which remained substantially impaired, suggesting that EC-Exo treatment markedly enhanced the gait of the mice (Figures 2D–G).

### 3.5 EC-Exo treatment improved the integrity of BBB in MCAO/R mice

**FIGURE 3:**
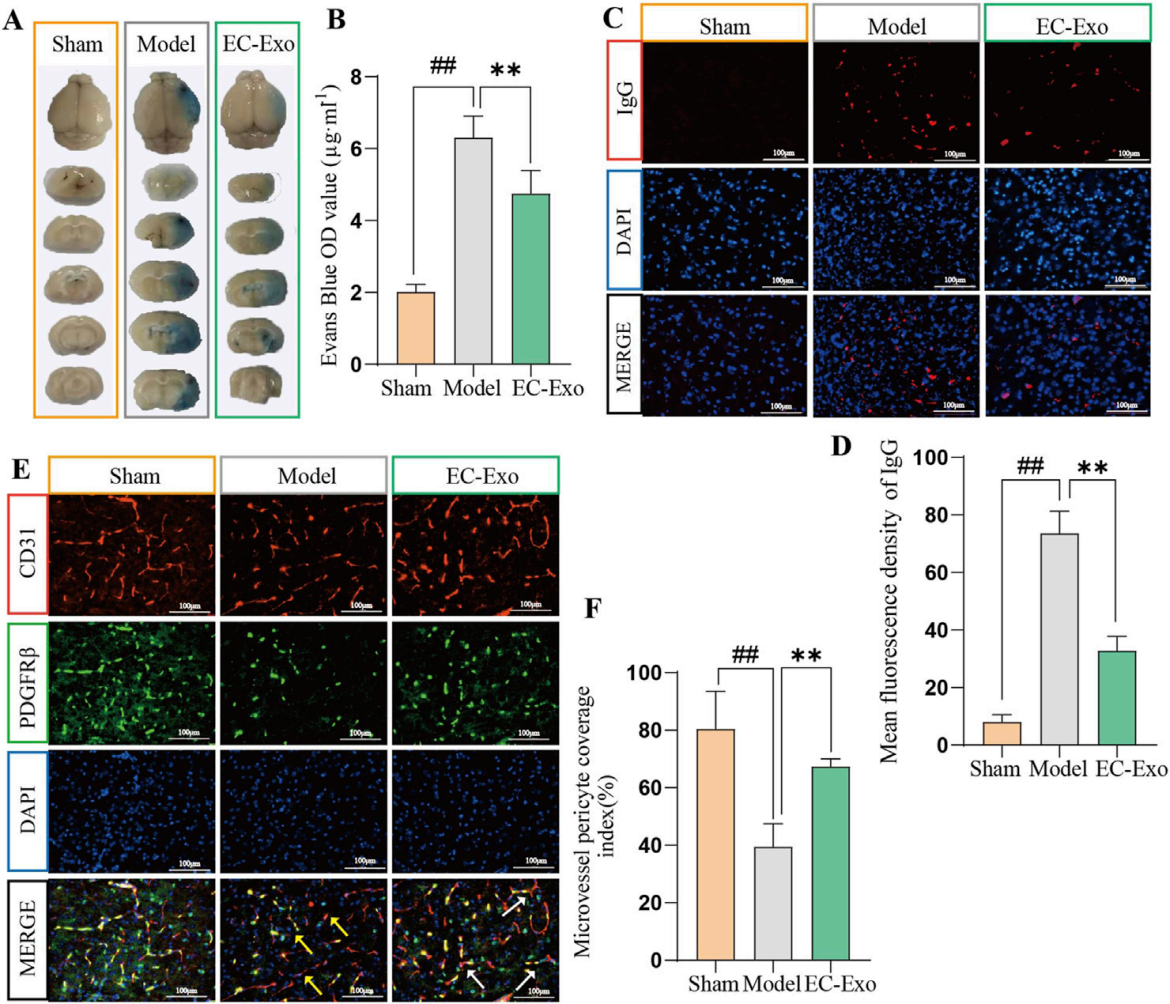
Effects of EC-Exo on BBB integrity in MCAO/R mice. **(A, B)** Representative images of EB extravasation in MCAO/R mice along with corresponding statistical findings (n = 4). **(C, D)** Representative images depicting IF staining and corresponding statistical analysis of IgG leakage in the ischemic cortex of MCAO/R (n = 4), Scale bar = 100 μm. **(E, F)** Representative images depicting IF staining and corresponding statistical analysis of CD31/PDGFRβ positive cells in the ischemic cortex of MCAO/R mice (n = 4), Scale bar = 100 μm. All data are expressed as mean ± SD (n = 14). The data were analyzed by one-way ANOVA followed by multiple comparisons using Tukey’s *post hoc* test. ^#^ *P* < 0.05, ^##^ *P* < 0.01 vs. control; ^*^ *P* < 0.05, ^**^ *P* < 0.01 vs. MCAO/R. Effects of EC-Exo on the integrity of BBB in MCAO/R mice. **(A–B)** The integrity of the BBB in mice was analyzed by Evans blue (n = 4) and IgG leakage **(B, C)** (n = 4), with a scale bar of 100 μm. **(E, F)** Effect of EC-Exo on pericellular coverage in MCAO/R mice. Representative images and statistical analysis of IF staining for CD31/PDGFRβ positive cells in the ischemic cortex of MCAO/R mice (n = 4), with a scale bar of 100 μm. The data were analyzed by one-way ANOVA followed by multiple comparisons using Tukey’s *post hoc* test. ^#^ *P* < 0.05, ^##^ *P* < 0.01 vs. control; ^*^ *P* < 0.05, ^**^ *P* < 0.01 vs. MCAO/R.

The integrity of the BBB in MCAO/R mice was evaluated using Evans blue dye extravasation analysis. The results indicated increased EB extravasation in the MCAO/R group. Moreover, EC-Exo treatment markedly reduced EB extravasation (Figures 3A, B). IF analysis revealed a significant increase in IgG extravasation in the model group, while EC-Exo treatment markedly reduced this leakage (Figures 3C, D). IF double staining for CD31/PDGFRβ revealed that the model group exhibited disrupted blood vessels, a marked reduction in PDGFRβ positive expression, and significantly decreased perivascular cell coverage compared to the sham operation group. EC-Exo intervention enhanced blood vessel morphology compared to the model group, resulting in more intact vessels and a significant increase in PDGFRβ positive expression and perivascular cell coverage (Figures 3E, F).

### 3.6 EC-Exo treatment promotes tight junction proteins in MCAO/R mice

**FIGURE 4:**
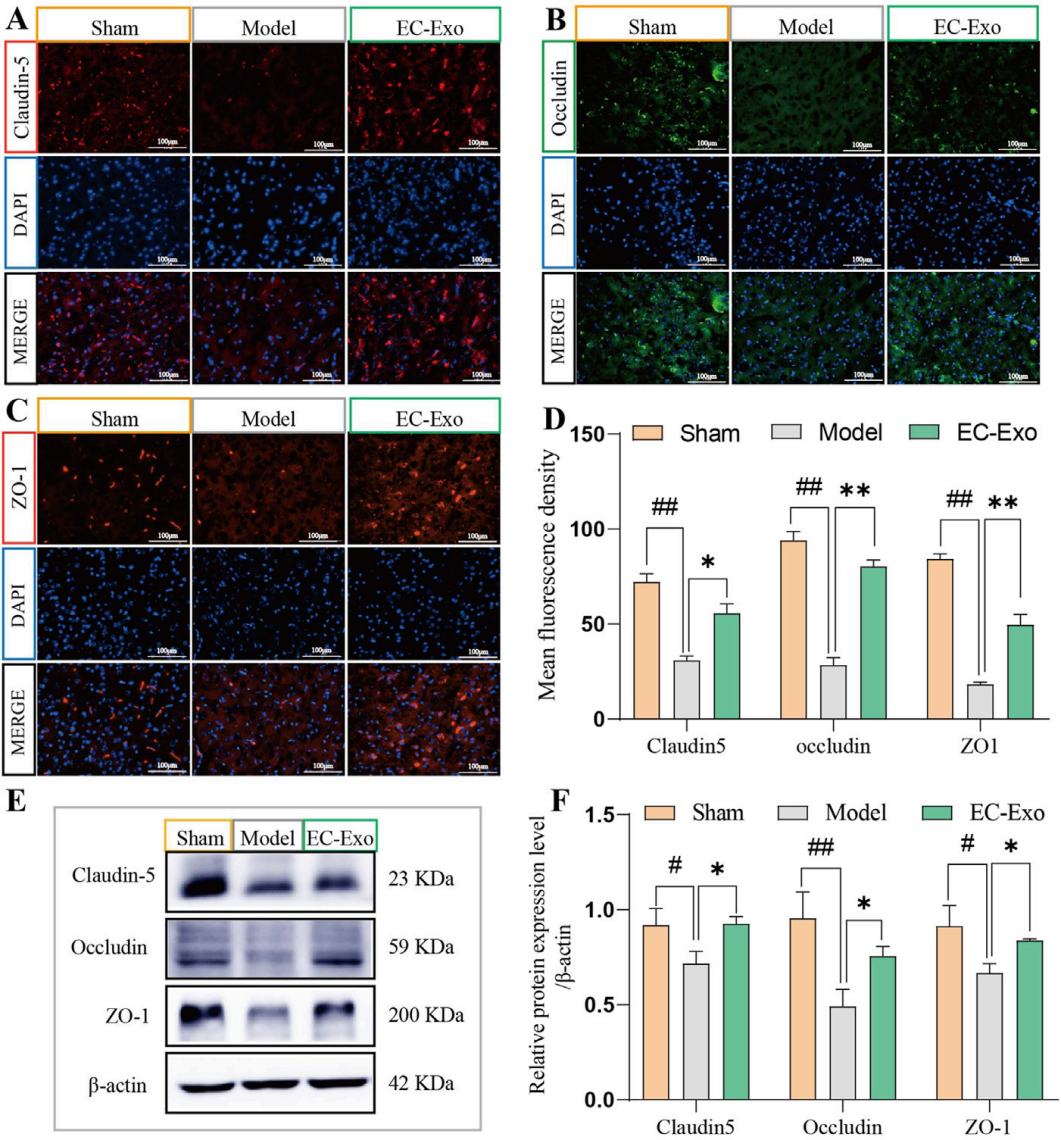
Impact of EC-Exo therapy on TJ proteins in MCAO/R mice. **(A–D)** Analysis of mean fluorescence intensity for tight junction proteins Claudin 5, Occludin, and ZO-1 in the cerebral cortex of mice using immunofluorescence staining (n = 4), with a scale bar of 100 μm. **(E, F)** Western blot analysis and quantification of Claudin 5, Occludin, and ZO-1 proteins (n = 4). The data were analyzed by one-way ANOVA followed by multiple comparisons using Tukey’s *post hoc* test. ^#^ *P* < 0.05, ^##^ *P* < 0.01 vs. control; ^*^ *P* < 0.05, ^**^ *P* < 0.01 vs. MCAO/R.

IF staining analysis revealed a significant reduction in the average fluorescence intensity of Claudin-5, Occludin, and ZO1 after ischemia induction compared to the sham group. EC-Exo treatment notably enhanced the average fluorescence intensity of Claudin-5, Occludin, and ZO1 for barrier tight junctions (*P* < 0.05) (Figures 4A–D). The Western blot analysis revealed a significant reduction in the expression of Claudin-5, Occludin, and ZO1 in the model group. Following EC-Exo intervention, there was a significant increase in the expression of Claudin-5, Occludin, and ZO1 proteins (*P* < 0.05) (Figures 4E,F).

### 3.7 EC-Exo treatment promotes basement membrane (BM) proteins in MCAO/R mice

**FIGURE 5:**
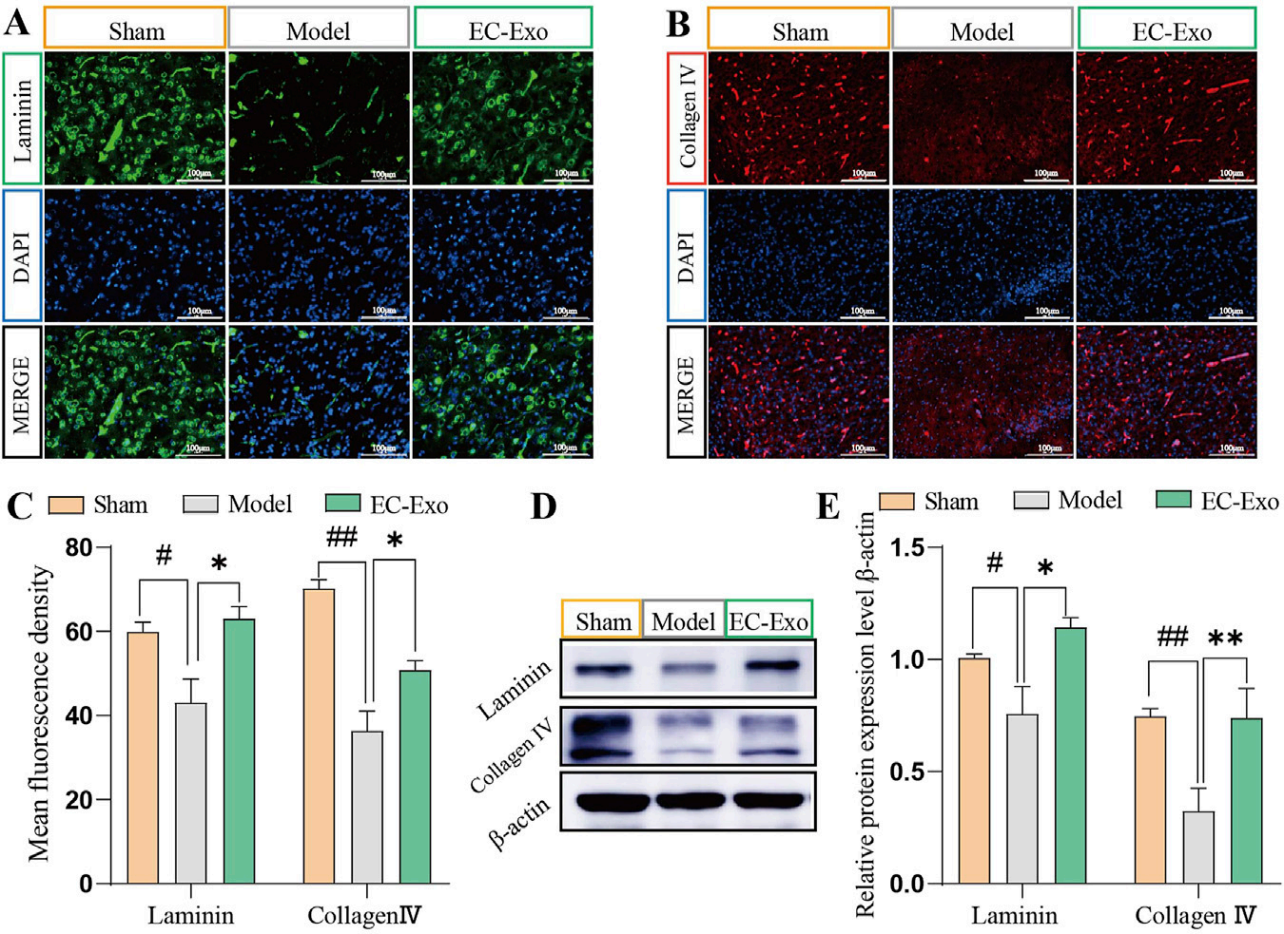
Effects of EC-Exo treatment on BM proteins in MCAO/R mice. **(A–C)** Analysis of the mean fluorescence intensity of BM proteins Laminin and Collagen IV in mice cerebral cortex by IF staining (n = 4), Scale bar = 100 μm. **(D–E)** Western blot analysis and quantification of Laminin and Collagen IV proteins (n = 4). The data were analyzed by one-way ANOVA followed by multiple comparisons using Tukey’s *post hoc* test. ^#^ *P* < 0.05, ^##^ *P* < 0.01 vs. control; ^*^ *P* < 0.05, ^**^ *P* < 0.01 vs. MCAO/R.

We assessed the impact of EC-Exo on BM remodeling by analyzing Laminin and Collagen IV expression in brain tissue using IF and Western blot. Statistical analysis revealed that compared to the sham group, the model group exhibited significantly reduced average fluorescence intensity of Laminin and Collagen IV (*P* < 0.05), while the EC-Exo group showed increased levels (*P* < 0.05) (Figures 5A–C). Western blot analysis revealed a significant reduction in Laminin and Collagen IV expression in the model group, with a notable increase following EC-Exo intervention (*P* < 0.05) (Figures 5D, E).

### 3.8 EC-Exo promotes PDGF-PDGFRβ and ang/tie-2 signaling pathways in MCAO/R mice

**FIGURE 6:**
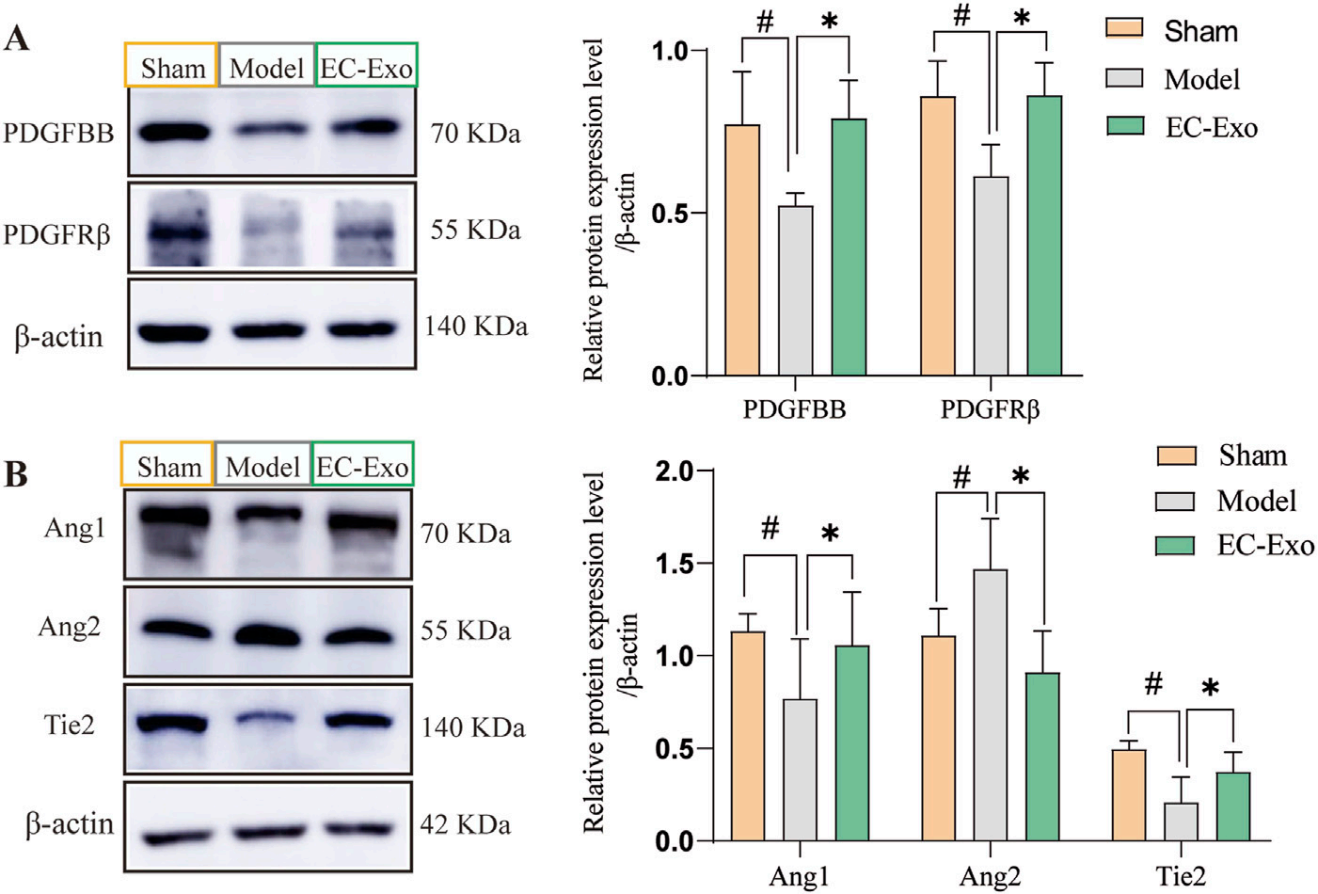
Effects of EC-Exo on PDGF-PDGFRβand Ang/Tie-2 signaling pathways in MCAO/R mice. **(A)** Western blot analysis of PDGF-BB, PDGFR-β, and β-actin, along with their quantitative measurements across groups. **(B)** Western blot analysis of Ang1, Tie2, and Ang2, along with their quantification across groups. (n = 4). The data were analyzed by one-way ANOVA followed by multiple comparisons using Tukey’s *post hoc* test. ^##^ *P* < 0.01 vs. control; ^*^ *P* < 0.05, ^**^ *P* < 0.01 vs. MCAO/R.

To further investigate the molecular signaling mechanism underlying BBB injury and angiogenesis promotion after cerebral ischemia in mice, we assessed the expression of the PDGF-PDGFRβ and Ang1/Ang2-Tie2 signaling pathways. In the model group, protein expressions of PDGFBB and PDGFRβ were notably reduced (*P* < 0.05). EC-Exo treatment notably elevated PDGFBB and PDGFRβ protein levels (*P* < 0.05) (Figure 6A). In the model group, there was a significant reduction in the protein expressions of Ang-1 and Tie-2. EC-Exo treatment markedly elevated Ang-1 and Tie-2 protein levels. The results demonstrated a significant increase in Ang-2 expression in the model group, which was subsequently significantly reduced following EC-Exo intervention (Figure 6B).

### 3.9 Inhibition of PDGFRβ reversed the effect of EC-Exo treatment on barrier integrity *in vitro*

**FIGURE 7:**
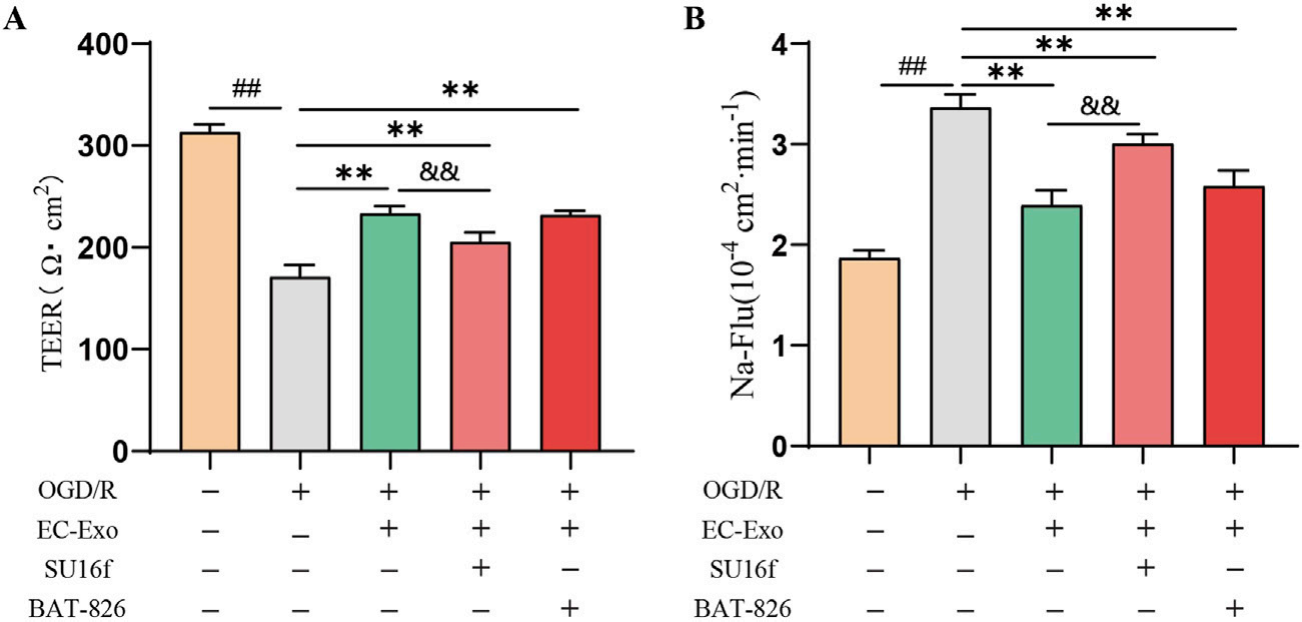
Effects of EC-Exo combined with PDGFRβ or Tie2 inhibitor on thebarrier integrity *in vitro* **(A)** The TEER values on the barrier integrity of co-culture system (n = 3). **(B)** The permeability of Na-Flu (n = 3). The data were analyzed by one-way ANOVA followed by multiple comparisons using Tukey’s *post hoc* test. ^##^ *P* < 0.01 vs. control; ^**^ *P* < 0.01 vs. OGD/R.^and&^ *P* < 0.01 vs. EC-Exo.

The PDGFRβ inhibitor (SU16f) and Tie2 inhibitor (BAY-826) into the *in vitro* BBB co-culture model to investigate their roles in barrier integrity mediated by EC-Exo. The results demonstrated that inhibition of PDGFRβ substantially diminished the protective effect of EC-Exo on BBB integrity. This was evidenced by significantly lower TEER values and increased Na-Flu permeability in the EC-Exo + SU16f group compared with the EC-Exo group. However, compared with the group treated with EC-Exo alone, the concurrent addition of BAY-826 to inhibit Tie2 expression did not significantly affect either the TEER value or the Na-Flu permeability (Figures 7A, B).

## 4 Discussion

The ischemic stroke is a neurological disorder characterized by inadequate blood and oxygen supply to the brain, leading to significant mortality and disability. Consequently, it has become a primary focus of research in terms of prevention and treatment (Alhadidi et al., 2022; Yang et al., 2022). Exosomes, a specific subtype of extracellular vesicle ranging in size from 30 to 150 nm, possess the ability to traverse the BBB. These exosomes are derived from diverse cells and organs located outside the central nervous system. They exhibit low immunogenicity and excellent biocompatibility, making them extensively investigated for their potential in promoting neural regeneration following ischemic stroke (Hong et al., 2019; Raposo and Stoorvogel, 2013; Lee et al., 2022). Research has shown that exosomes are a key mediator of intercellular communication, which participates in normal physiological processes and plays a role in the development and progression of disease (Meldolesi, 2018). Exosomes have great promise in the endogenous drug-delivery system of the BBB in cerebral ischemia, which is also an important basis for the treatment of ischemic stroke.

The isolation and purification of exosomes serve as the fundamental prerequisites for subsequent research endeavors. Exosomes, ranging in size from approximately 30–100 nm, are extracellular vesicles secreted by virtually all cell types and widely distributed throughout various bodily fluids. The isolation of exosomes was achieved using ultrafast centrifugation, and their presence was confirmed through particle size analysis, transmission electron microscopy, and protein labeling as recommended by the International Extracellular Vesicle Association (Witwer, et al., 2013). Therefore, we opted for the isolation of exosomes through ultracentrifugation, followed by their characterization using TEM and DLS analysis and Western blot. The TEM analysis revealed the presence of spherical structures with diameters ranging from 30 to 150 nm. Similarly, DLS particle size analysis indicated that the particle sizes fell within the range of 30–150 nm. Western blot analysis further confirmed the expression of exosome marker proteins TSG101, CD63, and CD9 in EC-Exo. Furthermore, the absence of Calnexin (a non-exosomal marker) in EC-Exo confirmed the minimal contamination from cellular debris or organelles. These comprehensive analyses substantiated that the obtained exosomes adhered to established standards.

Walking disability is a prominent motor function disorder that exerts a significant impact on stroke patients, with abnormal gait in this population being characterized by diminished stride length and reduced stride amplitude, among other distinctive features (Poongodi et al., 2024). The DigiGait analysis system represents a non-subjective and highly reproducible behavioral testing technology utilizing kinematic and biomechanical methods to investigate walking patterns, identify aberrant gait, and subsequently evaluate treatment outcomes (Akula et al., 2020). It has gained widespread utilization for evaluating the behavioral characteristics of the MCAO model in recent years. Impaired motor function in animals is typically characterized by reduced paw area and shortened stride length as reliable indicators. This study demonstrates that EC-Exo effectively enhances both paw area and stride length in MCAO/R mice, particularly improving motor function on the affected limb after surgery, thereby highlighting the beneficial impact of EC-Exo on ischemia-reperfusion-induced brain injury in mice.

The impairment of the BBB is a critical pathophysiological mechanism in ischemic reperfusion injury, leading to hemorrhagic conversion and exacerbation of stroke (Gao et al., 2023). Maintaining BBB integrity is crucial for treating cerebral ischemic reperfusion injury. Pericytes play a pivotal role in maintaining microvascular integrity and facilitating repair processes following ischemic stroke (Seyedaghamiri et al., 2024; Liu et al., 2012). In the aftermath of a stroke, pericytes exert reparative effects on injured blood vessels through coordinated interactions with endothelial cell-associated pathways and signaling factors. *In vitro* studies have shown the addition of EC-Exo inhibited pericyte lysis (increased pericyte density/coverage), increased cell TEER values and decreased Papp of Na-Flu in the endothelial-pericyte co-culture BBB system after OGD/R injury. Furthermore, the addition of GW4869 inhibitor to suppress endothelial cell exosome secretion resulted in the inhibition of these effects. In alignment with *in vitro* results, *in vivo* studies show that EC-Exo decreases Evans Blue dye permeability, reduces IgG leakage in the penumbra, and improves perivascular cell coverage on the ischemic cortex in MCAO/R mice. The results indicate that EC-Exo may improve motor nerve function by increasing perivascular cell coverage and strengthening vascular barrier integrity, underscoring the importance of exosomes in endothelial cell and pericyte communication.

The preservation of BBB integrity relies on the essential role of endothelial cell tight junctions (Langen et al., 2019; Willis et al., 2004). Following a stroke, disrupted endothelial cell tight junctions lead to increased permeability of the perilesional region, compromising BBB function (Qi et al., 2024). Tight junctions are complex structures composed of transmembrane proteins, including Claudins, occludins, zonula occludens 1 (ZO-1), and cytoskeleton-associated proteins. The basement membrane (BM) also plays a crucial role in maintaining the integrity of the BBB. Disruption of the BM structure results in detachment of pericytes from the BM and separation from brain microvascular endothelial cells, leading to compromised cell-cell junctions and increased permeability of the BBB (Yao, 2019; Abdullahi et al., 2018). Strengthening intercellular tight junctions between endothelial cells and reinforcing the basement membrane’s structural integrity are crucial for maintaining the BBB’s integrity (Zheng et al., 2023). The *in vivo* investigations have demonstrated that EC-Exo can effectively induce the expression of crucial TJ proteins, namely, claudin5, occludin, and ZO-1, as well as BM proteins including laminin and Collagen Ⅳ. This substantially enhances the structural integrity of the BBB in mice undergoing MCAO/R.

Various signaling pathways mediate the interaction between endothelial cells and pericytes, ensuring vascular stability and preserving the structural and functional integrity of the BBB. Research indicates that PDGF-PDGFRβ signaling is crucial for preserving vascular barrier integrity and stability. The secretion of PDGF-BB by endothelial cells represents the most potent regulatory mechanism for pericyte recruitment and retention within neovascular structures (Yao et al., 2022; Hori et al., 2004). Concurrently, pericytes exert regulatory control over endothelial cell proliferation, migration, and differentiation while also contributing to capillary stabilization through modulation of PDGFR-β. Furthermore, Ang-1/Ang-2 significantly influences angiogenesis and the maintenance of BBB integrity. The vascular endothelial receptor tyrosine kinase Tie2, which is an endogenous ligand of Ang1, is crucial for new blood vessel formation and regulation, significantly contributing to post-stroke recovery (Féraud et al., 2003; Hansen et al., 2008). Ang2, a growth factor in the Ang/Tie signaling pathway, plays a key role in angiogenesis (Gurnik et al., 2016; Lv et al., 2022). Activated pericytes can produce Ang-1, which promotes pericyte recruitment and stimulates endothelial cell secretion of PDGF-BB by binding to Tie 2 on endothelial cells (Hosaka et al., 2018; Cash et al., 2023). This study shows that EC-Exo promotes pericyte recruitment for neovascularization in ischemic stroke mice by activating PDGF-PDGFRβ and Ang1/Ang2-Tie2 signaling pathways, leading to neovascular maturation, improved stability, and enhanced BBB integrity. Further, the role of PDGFRβ inhibitors and Tie2 inhibitors in barrier integrity mediated by endothelial cell exosomes was investigated using an *in vitro* BBB co-culture model. The results demonstrated that inhibiting PDGFRβ expression markedly attenuated the enhancement of TEER values and the reduction in Na-Flu permeability induced by endothelial cell exosomes. In contrast, inhibition of Tie2 expression did not significantly affect either TEER values or Na-Flu permeability. However, the aforementioned findings only demonstrate the described effects *in vitro*. To validate these observations, further loss-of-function experiments, such as siRNA-mediated knockdown of PDGFRβ in brain endothelial cells *in vivo*, are indispensable. This aspect will be addressed in subsequent in-depth studies.

Compared with other types of exosomes, EC-Exo may offer higher brain-targeting efficiency due to the natural expression of endothelial cell-specific markers. This characteristic may be particularly valuable in diseases involving endothelial damage, such as ischemic stroke, while MSC-Exos are more commonly used in anti-inflammatory and tissue regeneration applications (Gao, et al., 2018; Babaei and Rezaie, 2021). In addition, given that EC-Exo are derived from endothelial cells, their surface proteome and receptor profiles exhibit high compatibility with those of endothelial cells. This feature is likely to mitigate the risk of immune rejection. Compared with traditional therapies, exosomes exhibit significant advantages over recombinant proteins or small molecule drugs. These advantages include high stability, low molecular weight, the ability to easily cross the BBB, and superior delivery efficiency, which collectively position EC-Exo as a promising tool for the treatment of central nervous system diseases.

## 5 Conclusion

This study shows that EC-Exo strengthens BBB integrity by facilitating perivascular cell migration, minimizing their dissociation, enhancing perivascular cell coverage, and upregulating tight junction and basement membrane protein expression in endothelial cells. Consequently, the aforementioned findings contribute to the understanding of how EC-Exo improves motor function in mice and exerts neuroprotective effects. Although our study demonstrates the protective role of EC-Exo on BBB integrity, the specific cargoes responsible for these effects remain to be fully characterized. Future profiling of EC-Exo (e.g., by mass spectrometry or sequencing) will help identify key mediators, such as miRNAs or proteins, that may synergistically regulate PDGFRβ/Tie2 signaling.

## Data Availability

The raw data supporting the conclusions of this article will be made available by the authors, without undue reservation.
